# The Impact of the COVID-19 Pandemic on Israeli Orthodontic Practice: A Clinic’s Activity and Patients’ Attitudes

**DOI:** 10.3390/ijerph19041965

**Published:** 2022-02-10

**Authors:** Tatiana Sella Tunis, Tal Ratson, Shlomo Matalon, Michael Abba, Alex Abramson, Moshe Davidovitch, Nir Shpack

**Affiliations:** 1 Department of Orthodontics, The Maurice and Gabriela Goldschleger School of Dental Medicine, Tel Aviv University, Ramat Aviv, Tel Aviv 69978, Israel; davidom@tauex.tau.ac.il (M.D.); nir@shpack.co.il (N.S.); 2 Department of Pediatric Dentistry, The Maurice and Gabriela Goldschleger School of Dental Medicine, Tel Aviv University, Ramat Aviv, Tel Aviv 69978, Israel; talratso@post.tau.ac.il; 3 Department of Oral Rehabilitation, Head, The Goldschleger School of Dental Medicine, Tel Aviv University, Ramat Aviv, Tel Aviv 69978, Israel; matalons@tauex.tau.ac.il; 4 Department of Oral and Maxillofacial Surgery, Barzilai Medical Center, Ashkelon 7830604, Israel; drabba@me.com (M.A.); alexa@bmc.gov.il (A.A.)

**Keywords:** COVID-19, orthodontics, lockdown, canceled appointments, emergency visits

## Abstract

The current study aimed to characterize the activity in orthodontic clinics during the COVID-19-induced lockdown and the inter-lockdown periods, as well as to evaluate patients’ perspectives with respect to their fears, their extent of cooperation with treatment, and their emergency needs during the lockdown. The data were gathered from 11 private orthodontic clinics from 1 January 2020 to 8 March 2021, which included three lockdowns and inter-lockdowns. Information specifying the number of admissions, missed appointments, and emergency visits was gathered. Four hundred and twenty-nine orthodontic patients treated in those clinics agreed to complete a questionnaire that evaluated their concerns and expectations, the treatment emergency issues, implementation of the orthodontist’s instructions, and contact with the clinical staff during lockdowns. There was a significant increase in the number of scheduled appointments during the inter-lockdown periods, compared with the pre-pandemic period (*p* = 0.001). No difference in the number of missed/canceled or emergency appointments was found between the different periods (*p* > 0.420). The majority (89.6%) of the emergency visits involved issues with appliances; 68.7% of the subjects were advised to present themselves at clinics. During the peaks of the waves of the COVID-19 pandemic, a sharp rise in the number of missed and urgent appointments was not found. Reducing the number of orthodontic emergencies may assist in reducing patient fears.

## 1. Introduction

On 11 March 2020, the World Health Organization (WHO) declared the coronavirus disease outbreak of 2019 (COVID-19) a global pandemic [1]. This disease is caused by severe acute respiratory syndrome coronavirus 2 (SARS-CoV-2); it rapidly spread throughout the world [2]. Owing to its rapid transmissibility (direct inhalation of airborne virus or indirect transfer to the mouth from surfaces contaminated with virus-laden salivary/respiratory secretions) [3,4], and the relatively high infection fatality rate (1.4–9.6%) [5], all social settings including medical service professions, especially dentistry, were significantly adversely affected. Public safety policy was promoted to limit dental and orthodontic care to only urgent/emergency treatment [4].

It was previously reported that an elevated level of COVID-19-related anxiety exists in the patient population, and that it is associated with increased fear of receiving needed dental care [6]. This was also reported among orthodontic patients, who preferred to postpone orthodontic treatment, and to avoid dental appointments in general due to the fear of contamination at the dental office [7,8]. On the other hand, surveys of patients undergoing orthodontic treatment during this period revealed that patients expressed anxiety over the lack of access to clinical care facilities and the resulting prolongation of treatment, which were found to be sources of related mental distress [8,9]. In addition, a significant level of distress was also found among dentists [10,11] and dental assistants [12]. A study of Italian dentists, including orthodontists, indicated that they were most concerned about the risk of contamination and consequently, they performed fewer emergency treatments. Whereas, all other dental procedures can be administered over a brief period, orthodontic treatment is a prolonged process due to the biological limitations inherent in tooth movement. Many such treatments can be considered elective; however, their proper administration requires prospective patient follow-up over time, as well as appliance adjustments (typically performed once every three to four weeks). The disturbance of routine daily activities and the imposition of lockdown conditions may affect the status of orthodontic patients and could challenge the orthodontist and clinic staff to provide proper dental treatment under pandemic conditions.

In Israel, the Ministry of Health regularly updates and informs the professional and general population by issuing new guidelines aimed to restrict the spread of the virus. Dentistry was required to discontinue its routine function during the first lockdown. However, Israel faced relatively few waves of the pandemic; the lockdown and inter-lockdown periods are already adequately described in general but without specifically analyzing orthodontics [13,14,15,16,17].

The COVID-19 pandemic has been ongoing for two years, and its culmination is unclear. Since pandemic waves and subsequent lockdowns are still possible, with the appearance of new virus strains, there is an urgent need to analyze its impact on clinical activity during this period. The purpose of this study was twofold: (1) to investigate whether differences exist in orthodontic clinical activity regarding the number of scheduled, missed, or canceled appointments, and the number of emergency treatments during the waves of the pandemic, and (2) to evaluate patient attitudes regarding their anxieties, their cooperation with treatment protocols, and their in-person contact with their doctor during the lockdown.

## 2. Materials and Methods

### 2.1. Study Sample

This study was carried out using data derived directly from orthodontic clinics detailing their activity. These data were collected from the appointment logs and patients’ registration cards available from the eleven Israeli orthodontic clinics. All the professionals who managed those clinics were staff members at the Department of Orthodontics, the Maurice and Gabriela Goldschleger School of Dental Medicine, Tel Aviv University, Israel. Additionally, a questionnaire was distributed among orthodontic patients, or responsible adults treated by the corresponding care specialists, in order to assess their attitudes. The research was approved by the institutional ethical review board of Tel Aviv University. Inclusion criteria were as follows: complete records of patients’ attendance, i.e., proper registration of all dental visits, including unscheduled/emergency visits, and missed appointments. All the subjects were administered treatment by the care specialist and were required to provide informed consent to participate in the present study. Exclusion criteria included incomplete records in the clinic’s log book, incomplete responses in the questionnaire, or subjects that have a limiting physical or mental condition.

### 2.2. Appointment Logs

These data were assessed for the following seven T-periods:

- T0—the pre-pandemic and pre-lockdown situation: 1 January–18 March 2020 (78 days);
- T1—the first lockdown (extreme lockdown): 19 March–7 May 2020 (50 days);
- T2—the first inter-lockdown period: 8 May–17 September 2020 (133 days);
- T3—the second lockdown: 18 September–17 November 2020 (61 days);
- T4—the second inter-lockdown period: 18 November–26 December 2020 (39 days);
- T5—the third lockdown: 27 December 2020–7 February 2021 (43 days);
- T6—the post-lockdown period: 8 February–8 March 2021 (29 days).

The data gathered from the appointment logs included the following information for each T-period separately:

1. The total number of appointments: a count of all scheduled appointments during the specified period;
2. The adjusted number of appointments: the number of scheduled appointments relative to the period duration; this was calculated by dividing the total number of appointments by the number of days for the specified period;
3. The number of missed appointments: a count of the missed and canceled appointments by either patients or orthodontists during a specified period;
4. The number of emergency visits: the number of unscheduled appointments for a specified period;
5. The percentage of missed appointments and emergency visits; they were calculated as the proportion of missed appointments and emergency visits from the total number of appointments in the specified period.

### 2.3. Questionnaire

**Figure 1:**
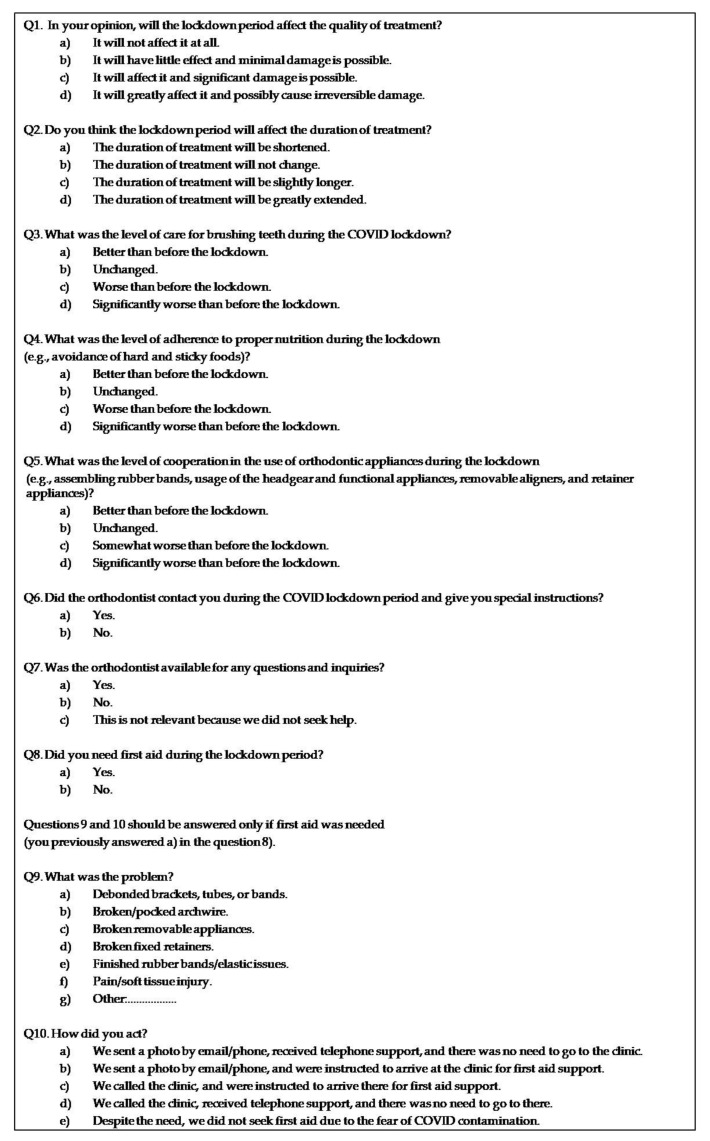
The questionnaire.

The participants were contacted during the first week of the T2 research period to obtain the data for the T1 period and were asked to complete the questionnaire. The questionnaire had a digital format; it was compiled using Google Forms and was sent to the patients electronically as a link using e-mail or WhatsApp Messenger. The questionnaire consisted of ten multiple choice questions (Q1–Q10) (Figure 1), encompassing demographics (age and sex), and the stage of treatment (up to 6 months in active treatment/more than 6 months in active treatment/towards the end of the treatment/the retention stage). The aim of the questionnaire was to evaluate four aspects of orthodontic treatment as perceived by the participating subjects:

- Cluster 1—Patients’ concerns and expectations (Figure 1, Q1–2);
- Cluster 2—Cooperation with the treatment protocol (Figure 1, Q3–5);
- Cluster 3—Contact with the orthodontist in dental care (Figure 1, Q6–7);
- Cluster 4—Emergency issues (Figure 1, Q8–10).

### 2.4. Validation of the Questionnaire

The questionnaire was evaluated by three experienced university-affiliated orthodontists, who were not directly or indirectly involved in the questionnaire design or the experimental process. In addition, two questionnaire construction experts evaluated the text for the presence of confusing, leading, or misleading questions. Consequently, a pilot study was undertaken utilizing 40 orthodontic patients from a single clinic. They were requested to specify if any question was unclear, and were encouraged to amend, or add the provided answers according to their understanding. Finally, the questionnaire was modified accordingly and finalized. The internal consistency of the questionnaire (the demographic part was omitted) was assessed with Cronbach’s alpha and the coefficient α = 0.92 was obtained.

### 2.5. Statistical Analysis

The data were recorded and analyzed using the IBM SPSS software package (Statistical Package for Social Sciences, version 20.0, SPSS, Inc., Chicago, IL, USA). All the quantitative measurements in the study were distributed normally. The assessment of normal distribution was based on a one-sample Kolmogorov–Smirnov test. The adjusted number of appointments (relative to the period duration), as well as the percentage of missed appointments and emergency visits, were compared between different periods using the related-samples Friedman’s two-way analysis of variance by rank test. The associations between patients’ age, treatment status, and sex were evaluated using the Mann–Whitney test. The Chi-Square test was run in order to determine the association between the patients’ responses and their sex/age group/treatment stage. The level of statistical significance was set at *p* < 0.05.

## 3. Results

### 3.1. Appointment Logs

**Figure 2:**
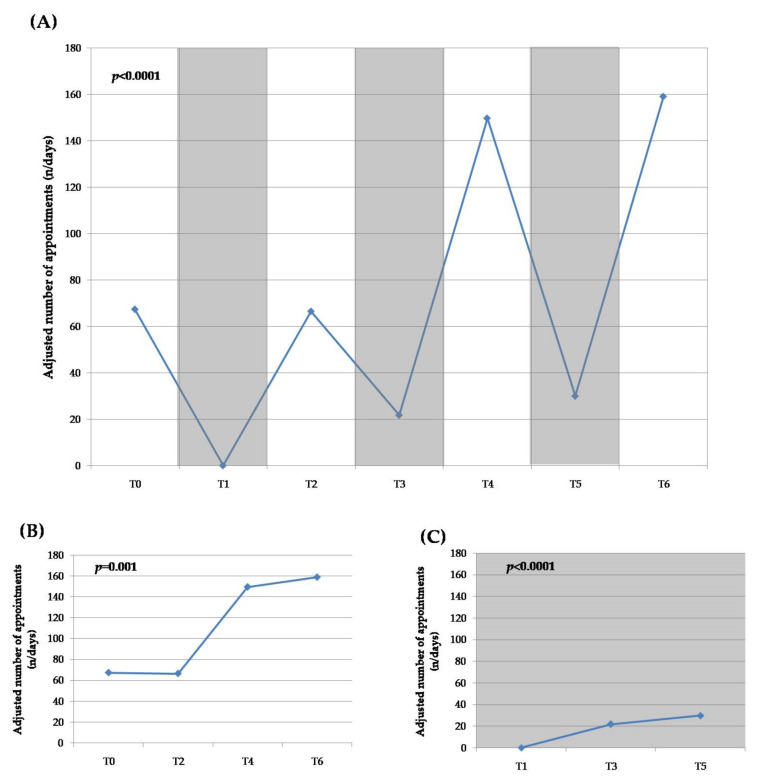
Graphical presentations of orthodontic clinical activity during the observation periods (T0–T6): (**A**) the adjusted number of orthodontic appointments for the total period; (**B**) the adjusted number of appointments for the inter-lockdown periods only; (**C**) the adjusted number of appointments for the lockdown periods only. Lockdown periods: T1, T3, and T5 (denoted in gray).

The total number of orthodontic appointments and their adjusted number differed significantly among the six subsequent periods (T0 and T2–T6; T1 was excluded due to its extreme nature) (*p* < 0.0001) (Table 1, Figure 2A). The adjusted number of appointments was found to oscillate in relation to the lockdown or inter-lockdown periods, displaying both decreased and increased clinical activity, respectively (Figure 2A). For the inter-lockdown periods, there was a significant increase in the number of appointments during the T4 and T6 periods, compared with T0 and T2 (*p* = 0.001) (Figure 2B). For the lockdown periods, there was a significant increase in the number of appointments during T3 and T5, compared with T1 (*p* < 0.0001) (Figure 2C). No statistically significant difference was found in the percentage of missed appointments and emergency visits between the six different periods (T1 was excluded) (*p* > 0.420) (Table 1).

### 3.2. Questionnaire

#### 3.2.1. Subjects’ Sex and Age

**Figure 3:**
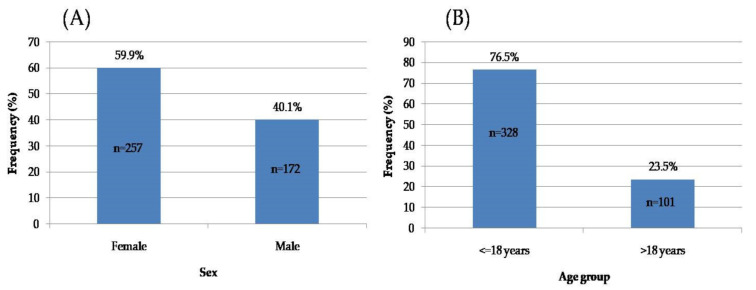
Distribution of the subjects’ sex (**A**) and age (**B**).

The study sample included 429 orthodontic patients: 172 males (40.1%) and 257 females (59.9%) (Figure 3A). The mean age was 19.23 ± 11.52 years (range: 6–75 years). The mean age did not differ significantly between the sexes; it was 18.95 ± 9.98 years for males and 19.42 ± 12.47 years for females (*p* = 0.06). Age was not normally distributed in both males and females (Kolmogorov–Smirnov test: *p* < 0.0001), and subjects were categorized into two age groups (≤18 years old/>18 years old) (Figure 3B).

#### 3.2.2. Subjects’ Treatment Stage

**Figure 4:**
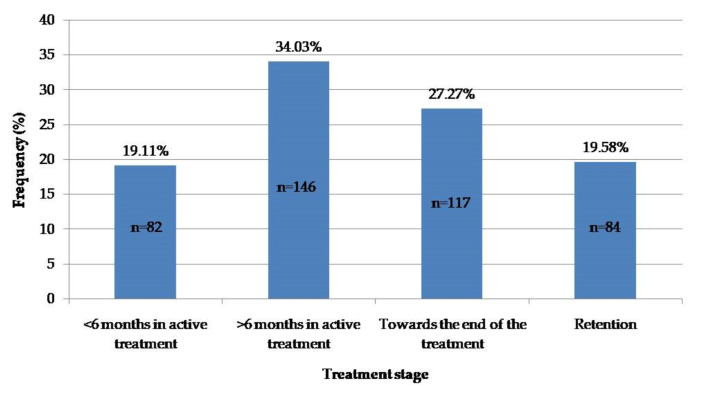
Distribution of the subjects’ treatment stages.

The majority of subjects were found to be in the active treatment stage; only 19.58% were in the retention stage (i.e., without fixed appliances) (Figure 4). No significant difference between the patient’s treatment stage and sex was found (*p* = 0.133).

#### 3.2.3. Subjects’ Responses

**Figure 5:**
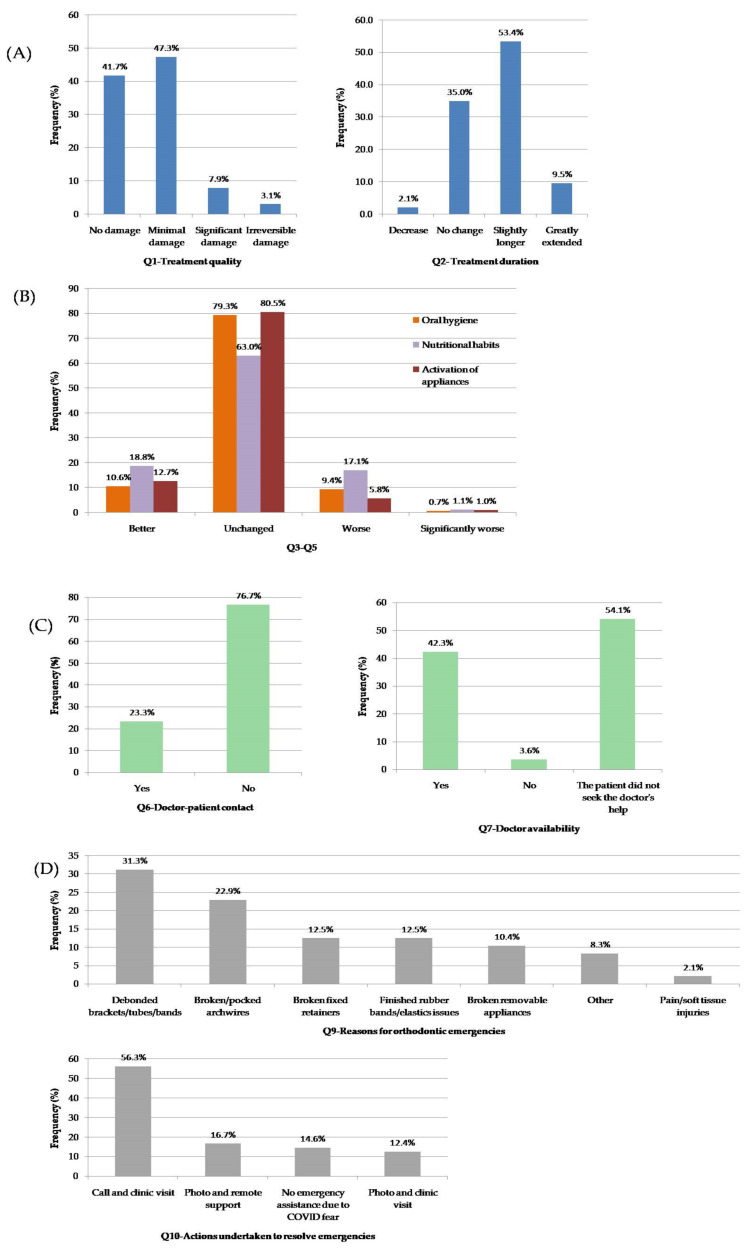
Bar charts presenting the subjects’ responses to the questionnaire, by clusters: (**A**) Cluster 1: Q1–2, (**B**) cluster 2: Q3–5, (**C**) cluster 3: Q6–7, and (**D**) cluster 4: Q9–10.

Subjects’ responses were summarized and presented in Figure 5.

Cluster 1 (Q1–Q2): Patients’ concerns and expectations (Figure 5A).

Q1: The findings indicated that 41.7% of the subjects expressed confidence that the lockdown would not negatively influence the quality of their treatment. Only 3.1% expressed a high degree of concern that their treatment quality will be greatly affected, including the risk of incurring irreversible damage. No significant differences in these parameters were found between males and females (*p* = 0.687). Additionally, no significant association was found between subjects’ attitudes regarding thetreatment quality and their age (*p* = 0.929), or the stage of the treatment (*p* = 0.088).

Q2: According to our sample, 35% of the subjects thought that the treatment duration will not be affected, and 9.5% thought that it will be greatly extended. No significant differences were found between male and female attitudes (*p* = 0.755), and no significant differences were found between different age groups (*p* = 0.087). In contrast, a significant association was found between the subjects’ treatment stage and their expectation/opinion regarding the influence of lockdowns on the treatment duration (*p* < 0.0001) (Figure S1A). The subjects that were more than six months into active treatment and those who approached the end of their treatment overwhelmingly responded that they expected the treatment duration to be extended, compared with those who were in treatment less than 6 months.

Cluster 2 (Q3–Q5): Cooperation with the treatment protocol (Figure 5B).

The findings indicate that the majority of the subjects thought that their compliance with oral hygiene, nutritional habits, and activation of orthodontic appliances did not change during the lockdown (79.3%, 63%, and 80.5%, respectively). Correspondingly, 10.1%, 18.3%, and 6.8% of the respondents reported that their oral hygiene, nutritional habits, and activation of orthodontic appliances, respectively, worsened during the lockdown. No statistically significant difference was found between male and female subjects (*p* > 0.103), age groups (*p* > 0.650), or subject treatment stages (*p* > 0.365). A significant association was found between the subjects’ treatment protocol cooperation (Q5) and their opinion regarding the impact that the lockdown will have on the treatment duration (Q2) (*p* = 0.001) (Figure S1B) and quality (Q1) (*p* = 0.012) (Figure S1C). The subjects who reported a significant decline in their cooperation, also indicated that their treatment was expected to be significantly affected and that there was a high likelihood that irreversible damage would occur, as well as having expectations of the treatment duration to be either greatly extended or shortened.

Cluster 3 (Q6–Q7): Contact with the orthodontist in dental care (Figure 5C).

Q6: It was found that during the first lockdown only 23.3% of the treating doctors contacted individual subjects and provided them with special instructions. No significant associations were found between the doctor’s contact and the patient’s sex (*p* = 0.570), or the treatment stage (*p* = 0.052). However, a significant association was found between the doctor’s contact and the subject’s age (*p* = 0.004) (Figure S1D), specifically, older subjects reported that doctors’ contact occurred more often during the lockdown than did younger subjects.

Q7: The unavailability of the doctor in care was reported by only 3.6% of the subjects. The rest of the respondents either did not seek a doctor’s assistance or reported that their doctor was available during the lockdown. No significant associations were found between the doctors’ availability and subjects’ sex (*p* = 0.186), age (*p* = 0.476), or the stage of treatment (*p* = 0.294).

Cluster 4 (Q8–10): Emergency issues (Figure 5D).

Q8: Only 11.2% of the respondents (*n* = 48) (30 females, 18 males) reported needing emergency assistance during the first lockdown. No significant difference was found between the subject’s sex (*p* = 0.639), age (*p* = 0.308), and the stage of treatment (*p* = 0.276). However, a significant association was found between the subject’s opinion regarding COVID-19’s influence on the quality of treatment and the need for emergency treatment during this period (*p* = 0.011) (Figure S1E). Those patients who needed emergency treatment during the lockdown expected that the quality of their treatment would be affected, and that significant or irreversible damage would be anticipated.

Q9: It was found that the main reason for emergency treatment during the lockdown was related to issues with the orthodontic appliances (89.6%). This included bracket debondings, poking wires, broken removable appliances, and broken fixed retainers (Figure 5D). Dental complications such as tooth pain or mobility and primary tooth exfoliation comprised 10.4% of these emergency appointments.

Q10: Slightly more than half of the reported emergencies (56.3%) were resolved by calling the clinic to schedule an appointment requiring physical presence. One third of the subjects who reported needing emergency treatment used self-taken dental photos that were delivered to the orthodontists; half of the subjects required a clinical visit (12.4%). The other half received remote support from their orthodontist (16.7%). The rest of the subjects decided to avoid any contact with their orthodontist due to a strong fear of COVID-19 contamination (Figure 5D).

## 4. Discussion

### 4.1. Orthodontic Clinics’ Activity and Patients’ Attitudes

The current study clearly shows that the COVID-19 pandemic imposed conditions that affected the pre-pandemic “normative” activity of orthodontic clinics in Israel. Namely, it caused “ups and downs” in the volume of activity. These vacillations mainly resulted from the Ministry of Health’s orders regarding how to operate during the various phases of the pandemic; specifically, every spike in the total number of COVID-19 patients in the general population was accompanied by a general lockdown. These spikes were found to cause significant reductions in the orthodontic clinical activity (the T1, T3, and T5 periods).

The initial reactions of the Orthodontic Society to the developments related to the evolving pandemic were expressed as serious concerns about the possible COVID-19 transmission from patients during clinic visits [18,19,20,21]. Hence, the Israeli Ministry of Health issued an order to totally discontinue orthodontic clinical activities during the first lockdown (T1). This is the reason for the “zero” activity, reflected in the lack of appointments during this period. During this period (T1), very few of the clinics provided emergency care; 88 such visits reported from all the clinics are represented in the current study. Owing to strict profession-specific, risk reduction, anti-infection procedural changes, some operational capacity during the T3 and T5 lockdown periods was reported; however, this was mostly limited to patients who had urgent or complicated treatments that had to be closely monitored. Following a reduction in COVID-19 morbidity in the general population, the Ministry of Health rescinded nationwide lockdown orders; it permitted medical care professions to provide service under strict guidelines. In this study, these periods were observed as peaks in the clinics’ activity (T2, T4, and T6 periods).

In contrast to previous reports [8,9,18,19,22,23,24,25,26], the findings of the current study indicate that orthodontic clinics and patients have adjusted well to the conditions imposed by the pandemic. This is supported by the findings of increasing activity during both the lockdown and inter-lockdown periods. This was most evident during periods T4 and T6, compared with T2 and T0 (pre-pandemic) periods. The relative number of orthodontic appointments during these inter-lockdown periods almost doubled, compared with those during the pre-pandemic period. During this time the general population was required to wear facemasks in order to prevent the spread of COVID-19. It can be speculated that the wearing of masks in public places may have reduced any perceived stigma associated with having fixed orthodontic appliances. Therefore, this may explain the reported increased clinical activity, which may reflect a reduction in any stigma. However, this speculation requires further investigation in order to be substantiated. Previous studies concluded that the transmission rate of COVID-19 within a dental clinical setting is significantly lower than that reported within the general population [27,28]. This is probably due to the strict compliance with infection control guidelines, adhered to and enforced by the clinic staff members. The reduced risk to both orthodontic staff and patients during the treatment might also explain the increased clinical activity reported in this study. Additionally, in our study no significant difference was found in the percentage of missed and canceled appointments between the lockdown and the inter-lockdown periods in Israeli clinics. This finding may indirectly support lower levels of doctor and patient anxiety due to the pandemic in relation to orthodontic treatment. Previous studies [9,29,30] reported that more than a third of orthodontic patients are not concerned about the impact of the lockdown on their treatments, which is most likely related to their confidence in their orthodontist. The present study found that a majority of subjects reported either no significant or only minor concerns with respect to the length and quality of their orthodontic treatment during the lockdown period. Only about 10% of subjects reported that they thought that their treatment would be extended and accompanied by irreversible damage. In contrast to Cotrin et al. [9] and Valekar et al. [30], we did not find any significant difference between male and female subjects with respect to their fears. However, a significant difference was found between subjects’ fears and the stage of their treatment. Namely, patients that started treatment less than six months before the onset of the pandemic reported that they did not worry about their expected treatment duration, compared with those who were in the middle or towards the end of their treatment, who reported being concerned that their treatment would likely be prolonged.

In our study, the majority of subjects (63–80.5%) reported an unchanged attitude towards compliance with proper oral hygiene, cooperation with appliance management, and nutritional habits during the pandemic. The rest of the patients were divided almost equally between those who reported an improvement and those who reported declining cooperation. Similarly, a previous study reported that about 75% of subjects followed their doctor’s instructions regarding oral hygiene maintenance and diet, yet about a third reported they did not wear their removable appliance during the lockdown [30]. It is reasonable to assume that the stay-at-home circumstances can offer more opportunities and time for oral hygiene; however, only 10.6–18.8% of the subjects reported an improvement in their cooperation. Furthermore, we discovered a significant association between subject cooperation and their expression of anxiety regarding expectations related to their treatment. Those who reported declined cooperation expected that the lockdown would have a detrimental impact on the treatment quality and duration. Sangalli et al. [31] showed that remote monitoring during orthodontic treatment improves patients’ plaque control and reduces the onset of carious lesions. Thus, continuous follow ups and remote support may play an important role in improving patients’ cooperation and subsequently, may influence their attitudes regarding treatment success.

### 4.2. Orthodontic Emergencies and Doctor Availability

Our findings indicated that the number of emergency visits ranged between 3.3% and 4.8% of the total visits, and that they did not significantly differ between the lockdown and inter-lockdown periods. Interestingly, 11.2% of the orthodontic patients reported that they needed some kind of orthodontic assistance during the first lockdown. Quan et al. [32] reported that in a Chinese population, about 33.67% of orthodontic patients reported encountering orthodontic-related problems during the pandemic. In the present sample, about 90% of the subjects reported experiencing technical problems with their orthodontic appliances (e.g., debonded brackets, poking wires, and broken appliances) as the main reason for an emergency, and only about 10% had dental problems such as tooth mobility and pain. Similarly, Turkistani [25], Popat et al. [33], and Cotrin et al. [24] previously reported that the most common reasons for orthodontic emergencies were debonded brackets/tubes and poking wire.

In the present study, in about half of the emergencies reported, the subjects called their treating doctor (via a video or audio call), and one third sent a clinical photograph (using a message) to describe what had happened to them. These reports are in accordance with the recommendations that the initial assistance should be virtual (a photo/video/call) in order to confirm the emergency and reduce the need for a possible unscheduled appointment [18,29]. These findings are similar to those reported by Quan et al. [32] who found that 55.1% of their subjects contacted their attending doctor remotely, 23.14% of the subjects managed to resolve the problem on their own, and about one third deemed the problem tolerable; therefore, they did not take any specific measures.

Our findings indicate that in the majority of the orthodontic emergency situations, the subjects had to be physically present in the clinic to resolve their problem. This finding can be attributed to the emergencies being associated with damage to the appliance, thus requiring that the subjects present themselves to the doctor in care to rectify the problem. It was found that only 17% of the subjects reported the need for emergency treatment and that remote support was sufficient. Our findings suggest that although remote orthodontic teleassistance is an important tool for treatment monitoring and emergency assistance, generally, the subject’s physical presence is required to solve emergency orthodontic problems. Furthermore, we found an association between subjects seeking emergency treatment during lockdowns and the attitude that the quality of their treatment could be significantly adversely affected. Similarly, it has been previously reported that subjects who encountered orthodontic emergencies during the pandemic exhibited a significantly higher level of anxiety than those who did not [32]. Thus, the prompt and efficient resolution of orthodontic emergencies could provide beneficial effects regarding patients’ attitudes and wellbeing, and could reduce their levels of anxiety during the pandemic.

## 5. Limitations of the Study

All data for the present study were gathered from multiple clinics of a single country. Therefore, the data may not be applicable to other geographical regions. The use of self-reporting surveys of patients/subjects also has inherent tendencies to interject participant bias. The data gathered from the appointment logs regarding the number of emergencies during the lockdowns may have information bias (the underestimation rate), since some patients could avoid emergency assistance due to their fears and COVID-19 concerns during these periods.

## 6. Conclusions

During the current COVID-19 pandemic, numerous countries around the world experienced more than one lockdown; this has a high likelihood of recurring. Based on the results of the present study, the following conclusions can be drawn:

1. Orthodontic clinics were able to operate at their full capacity between periods of lockdowns but needed to adhere to strict measures to reduce the risk of infection to both patients and staff.
2. No significant change in the number of missed and canceled appointments was found during the pandemic.
3. A majority of orthodontic patients believed that the length and quality of their treatment may be slightly affected due to the pandemic, and a small minority (10%) expected that significant and irreversible damage would occur, in addition to an extended treatment duration.
4. The stage of the treatment was associated with patients’ anxieties and expectations; namely, the patients who approached the end of their treatment exhibited greater anxiety regarding the possibility of an extended treatment time.
5. A significant increase in the number of orthodontic emergency appointments during lockdowns was not found in the current study.
6. The remote monitoring of patients using various telecommunication tools is helpful, but it was found to be insufficient to manage the majority of the treatment emergencies. The main reason for unscheduled appointments was technical issues with an orthodontic appliance, which preferably should be solved by an expert within a clinic.
7. Reducing the occurrence of orthodontic emergencies may reduce patients’ fears and may provide patients with greater motivation for improved cooperation during the lockdown period.

## Figures and Tables

**Table 1:** Orthodontic appointments during the pandemic.

T-Period	Duration (Days)	Appointments (n)	Adjusted Appointments (n/Days)	Missed Appointments (% *)	Emergency Visits (% *)
T0	78	5252	67.33	539 (10.3%)	238 (4.5%)
T1	50	0	0	0	88
T2	133	8842	66.48	813 (9.2%)	326 (3.7%)
T3	61	1325	21.72	114 (8.6%)	58 (4.4%)
T4	39	5836	149.64	554 (9.5%)	194 (3.3%)
T5	43	1287	29.93	129 (10.0%)	62 (4.8%)
T6	29	4611	159.00	402 (8.7%)	158 (3.4%)

The results are presented for the period 1 January 2020 until 8 March 2021. Lockdown periods: T1, T3, and T5 (are denoted in gray). Adjusted appointments = the number of appointments for a given period/the number of days for that period. * The percentage was calculated by dividing the number of missed appointments or emergency visits by the number of appointments (n) during the given period*100.

## Data Availability

The data sets analyzed during the current study are available from the corresponding author on request.

## Supplementary Materials

The following supporting information can be downloaded at: https://www.mdpi.com/article/10.3390/ijerph19041965/s1, Figure S1: Graphic presentation of associations between subject responses: (A) Treatment duration (Q2) and the treatment stage of the subject; (B) treatment duration (Q2) and compliance with appliance activation (Q5); (C) treatment quality (Q1) and compliance with appliance activation (Q5); (D) doctor-patient contact (Q6) and patient age group; (E) treatment quality (Q1) and the need for an emergency visit during the lockdown (Q8).
