# Supplementary material for: The Impact of the COVID-19 Pandemic on Israeli Orthodontic Practice: A Clinic’s Activity and Patients’ Attitudes
Source: Int J Environ Res Public Health. 2022 Feb 10;19(4):1965. doi: 10.3390/ijerph19041965 (PMC8872275; doi:10.3390/ijerph19041965)
Supplement: Supplementary file 1 [file ijerph-19-01965-s001.zip › ijerph-1564569-supplementary.pdf]

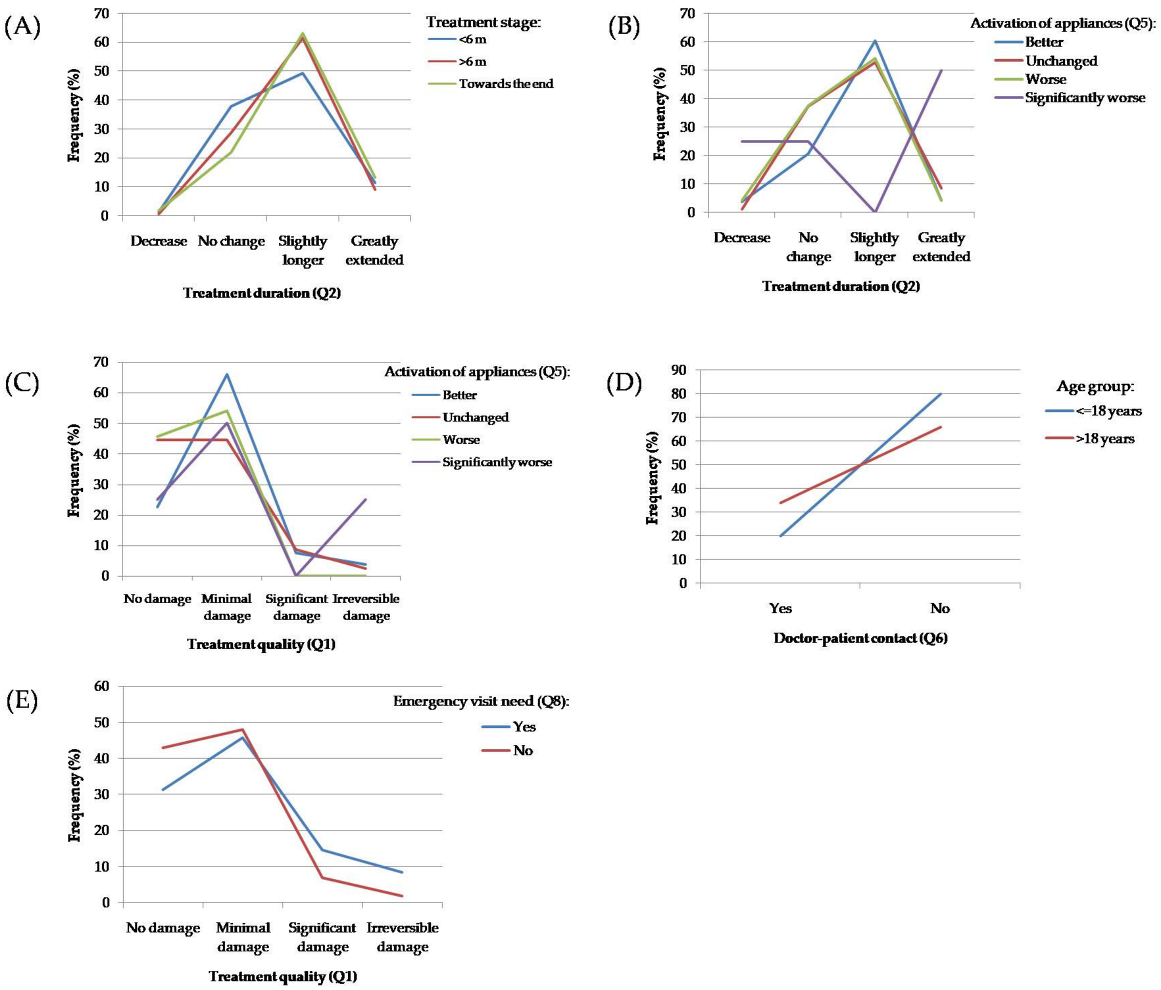

Figure S1. Graphic presentation of associations between subject responses: (**A**) Treatment duration (Q2) and the treatment stage of the subject; (**B**) treatment duration (Q2) and compliance with appliance activation (Q5); (**C**) treatment quality (Q1) and compliance with appliance activation (Q5); (**D**) doctor-patient contact (Q6) and patient age group; (**E**) treatment quality (Q1) and the need for an emergency visit during the lockdown (Q8).
